# Errata

**Published:** 2015-07-10

## Vol. 64, No. 19

In the report, “State-Specific Prevalence of Current Cigarette Smoking and Smokeless Tobacco Use Among Adults Aged ≥18 Years — United States, 2011–2013,” errors occurred. On page 533, in Table 1, the relative percent change (RPC) for cigarette smoking for Alabama (AL) should be −**11.5** and the RPC for smokeless tobacco for Washington (WA) should **not be marked as significant**. In addition, on page 534, in Figure 2, there was **no significant change** in smokeless tobacco in the District of Columbia.

## Vol. 64, No. 24

In the report, “State Tobacco Control Program Spending — United States, 2011,” an error occurred. The first sentence under the “What is added by this report?” summary, should read as follows:

In fiscal year 2011, for tobacco prevention and control activities, all 50 states and the District of Columbia combined spent $658 million ($2.11 per capita) in the following categories: 41.4% on state and community interventions ($272 million [$0.87 per capita]); 18.8% on health communication interventions ($124 million [$0.40 per capita]); 20.4% on cessation interventions ($134 million [$0.43 per capita]); 9.3% on surveillance and evaluation ($61 million [$0.20 per capita]); and 10.1% on **administration and management** ($67 million [$0.21 per capita]).

